# Supplementary material for: ZFHX3 Promotes the Proliferation and Tumor Growth of ER-Positive Breast Cancer Cells Likely by Enhancing Stem-Like Features and MYC and TBX3 Transcription
Source: Cancers (Basel). 2020 Nov 18;12(11):3415. doi: 10.3390/cancers12113415 (PMC7698617; doi:10.3390/cancers12113415)
Supplement: Supplementary file 1 [file cancers-12-03415-s001.pdf]

cancers

Supplementary Materials

ZFHX3 promotes the proliferation and tumor growth of ER-positive breast cancer cells likely by enhancing stem-like features and *MYC* and *TBX3* transcription

Ge Dong, Gui Ma, Rui Wu, Jinming Liu, Mingcheng Liu, Ang Gao, Xiawei Li, Jun A, Xiaoyu Liu, Zhiqian Zhang, Baotong Zhang, Liya Fu and Jin-Tang Dong

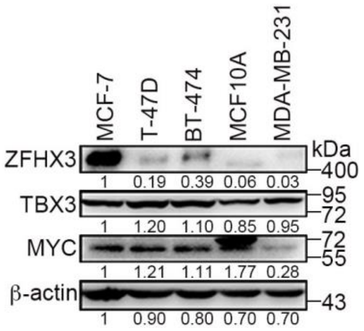

Figure S1. Expression of ZFHX3, MYC, and TBX3 in breast cell lines. The expression was detected by western blotting in breast cancer cell lines MCF-7, T-47D, BT-474, and MDA-MB-231 and immortalized non-tumorigenic human breast epithelial cell line MCF10A. Ratios of protein band intensities to those of their loading controls, with the control sample's normalized to 1, are shown under western blot bands. Uncropped western blot images are available in Supplementary Figure S10.

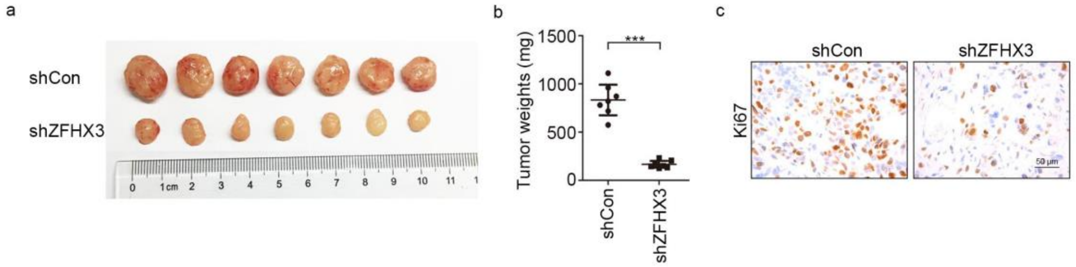

Figure S2. *ZFHX3* silencing in MCF-7 breast cancer cells attenuates tumor growth. (a,b) Tumor images (a) and weights (b) of MCF-7 cells with *ZFHX3* silencing. (c) Immunohistochemical staining of Ki67 in xenograft tumors of MCF-7 cells with *ZFHX3* silencing. The shZFHX3-1 shRNA was used to silence *ZFHX3*. shCon, control shRNA. *** $p < 0.001$.

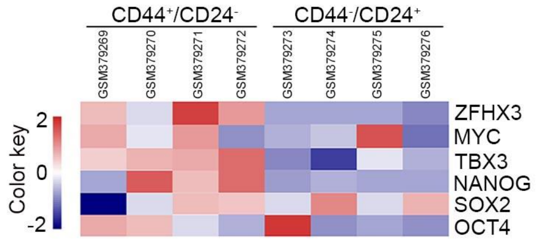

Figure S3. Association of ZFHX3 expression with those of BCSC markers in different populations of MCF10A cells. Expression evaluation of ZFHX3 and BCSC markers MYC, TBX3, NANOG, SOX2, and OCT4 between CD44+/CD24- and CD44-/CD24+ populations of MCF10A cells by heat map analysis in the GEO dataset GSE15192. Genes upregulated are marked red, and those downregulated marked blue. Data were from four replicates for each population.

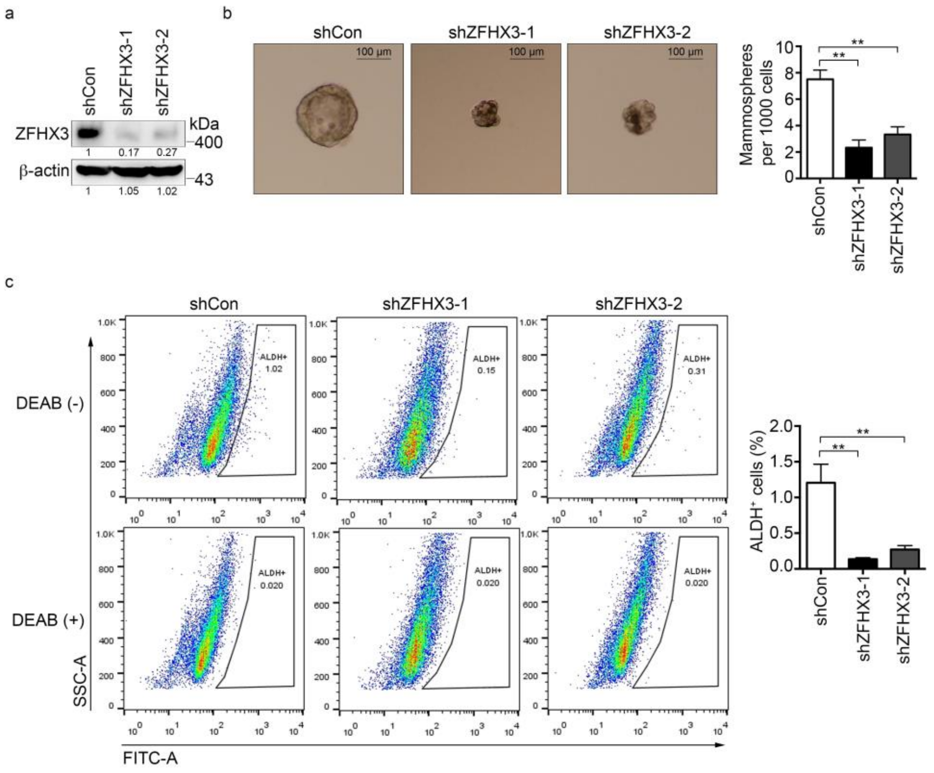

Figure S4. Stable knockdown of *ZFHX3* attenuates stem cell features in MCF-7 cells. Knockdown of *ZFHX3* by lentiviral shRNA in MCF-7 cells, which was confirmed by western blotting (a), reduced mammosphere formation, as indicated by images (b, left) and numbers (b, right) of mammospheres, and ALDH+ cells, as analyzed by flow cytometry (c). Ratios of protein band intensities to those of their loading controls, with the control sample's normalized to 1, are shown under western blot bands (a). ** $p < 0.01$. Uncropped western blot images are available in Supplementary Figure S11.

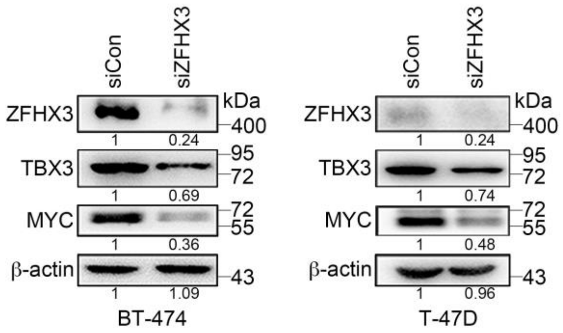

Figure S5. *ZFHX3* silencing reduces MYC and TBX3 expression in BT-474 and T-47D cells. Protein expression was determined by western blotting. Ratios of protein band intensities to those of their loading controls, with the control sample's normalized to 1, are shown under western blot bands. Uncropped western blot images are available in Supplementary Figure S12.

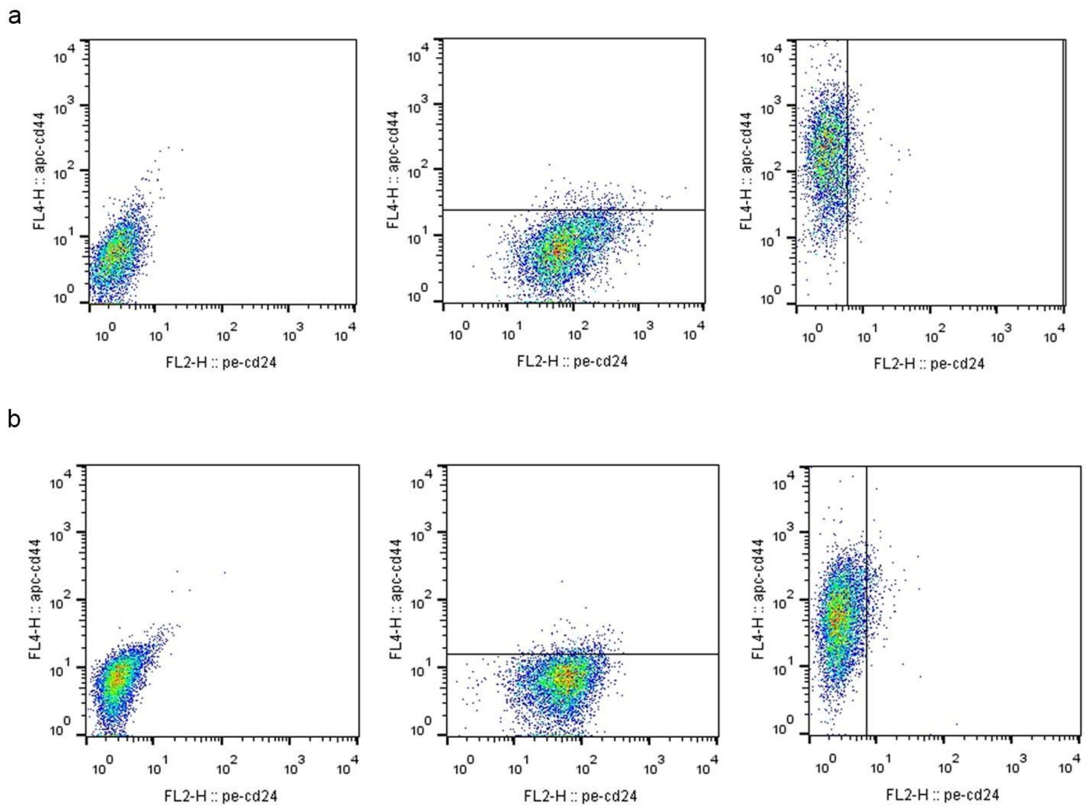

Figure S6. The blank and isotype controls for the flow cytometry analysis. The blank (left) and isotype controls for CD24 (middle, PE-anti-CD24) and CD44 (right, APC-anti-CD44) are shown for MCF-7 (a) and T-47D (b) cells. The gates for MCF-7 cells in Figure 2c and T-47D cells in Figure 2f were based on these blanks and isotype controls.

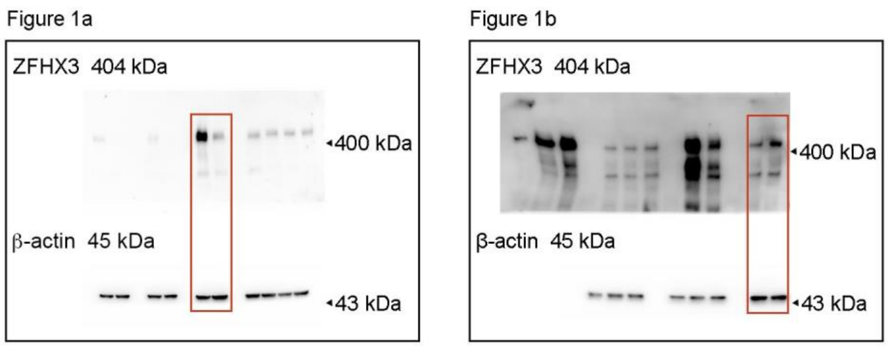

Figure S7. Uncropped western blot images for Figure 1.

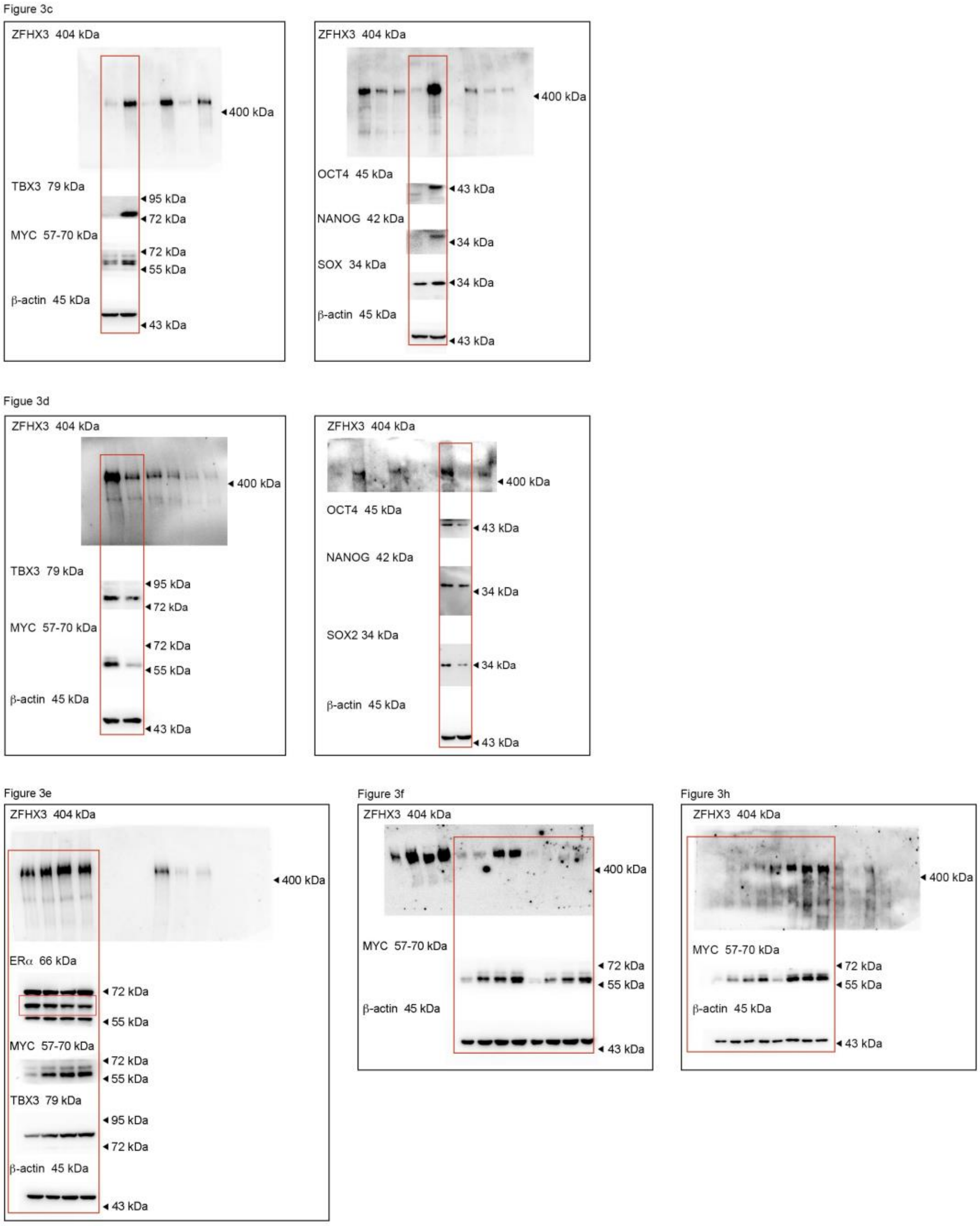

Figure S8. Uncropped western blot images for Figure 3.

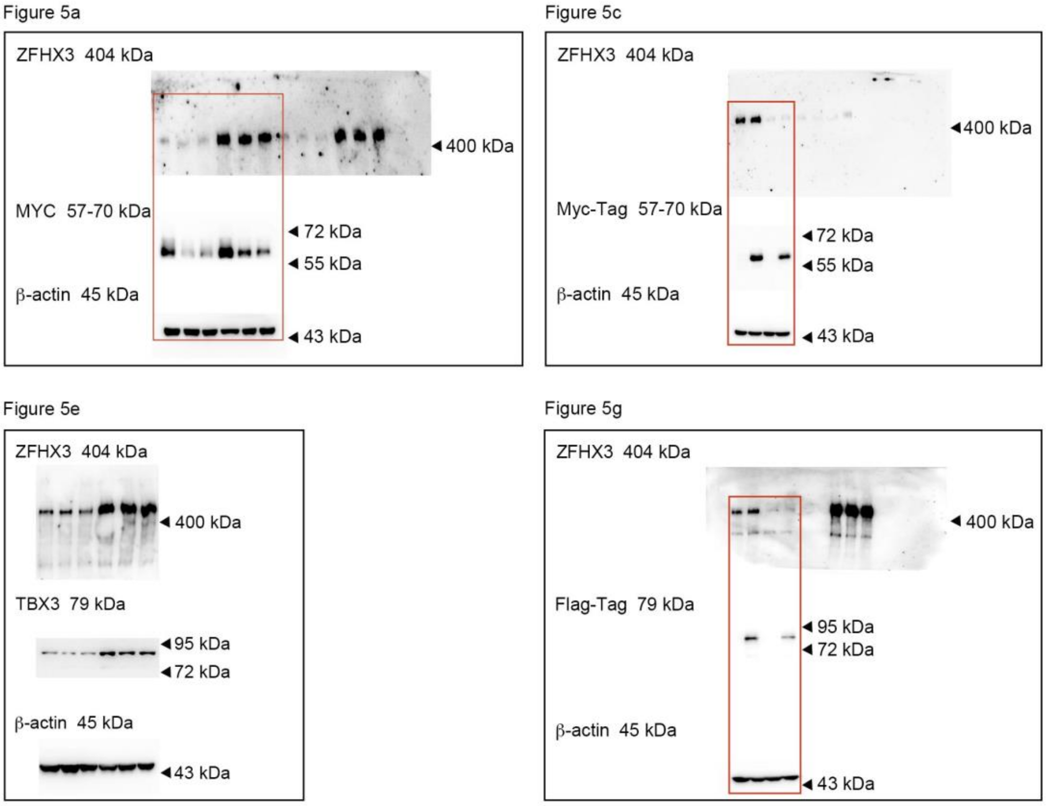

Figure S9. Uncropped western blot images for Figure 5.

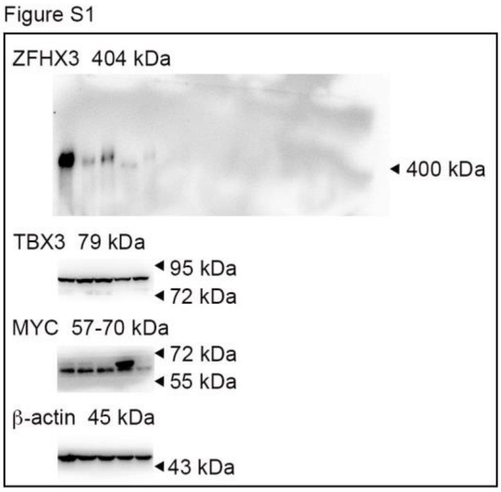

Figure S10. Uncropped western blot images for Figure S1.

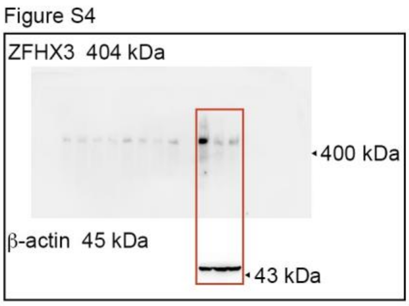

Figure S11. Uncropped western blot images for Figure S4.

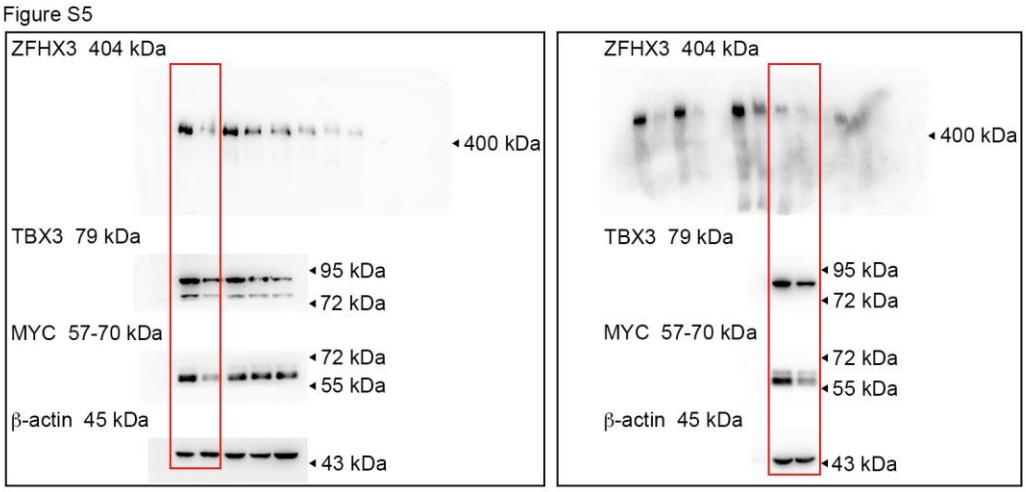

Figure S12. Uncropped western blot images for Figure S5.

Table S1. Primer sequences.

Gene	Primer sequences (forward/reverse, 5′–3′)
For RT-PCR:	
GAPDH	GGTGGTCTCCTCTGACTTCAACA/GTTGCTGTAGCCAAATTCGTTGT
ZFHX3	TGTTCCAGATCGAGATGGGAAT/CTTTCCCAGATCCTCTGAGGTTT
MYC	CTTTCCCAGATCCTCTGAGGTTT/TCAAGAGGTGCCACGTCTCC
TBX3	TGGGGACCTCTGATGAGTCCT/CCATGCTCCTCTTTGCTCTC
For luciferase reporter plasmids:	
MYC-Luciferase	CCGAGCTCAGGGAAAGACGCTTTGCA/GGAAGATCTGCGGTCACCATCTCCAG
MYC-B-Luci	CCGAGCTCGCAACTAGCTAAGTCGAAGC/GGAAGATCTGCCGTTCAGAGCGTGGGAT
ΔMYC-Luci	CCTTTTAGGGGAGGCGTGGGGGTGGGACGGT/CAACAGTACCGGAATGCCAAGCTTTCGCTGGAATTACTACAGCGAGTT
L-TBX3-Luci	CTAGCTAGCGAAACCCTGCAGTGACTTCCG/GGAAGATCTGCTCGAAATAGACACTCCAGC
S-TBX3-Luci	CTAGCTAGCGCGAGCGGAGTGCAAGAGAGG/GGAAGATCTCGGCGGCTCTAGAAGGTCG
For hIP-PCR:	
A (MYC)	GCGGGTTACATACAGTG/CCTCTTTCCCCTTTTATT
B (MYC)	ACCTGGAAAGGAATTAAACG/CTAAAAGGGGCAAG
C (MYC)	GAAGTCCGGTCCCGCGG/ACACGGAGTTCCCAATTTC
D (MYC)	GGGAGGCGTGGGGGTGGG/ATTGGATACCTTCCACCC
a (TBX3)	CGGCGGCCGGGCGGA/GCTCGCCTCTCCGACCG
b (TBX3)	GGAGTGCAAGAGAGGCGAGC/CTCGCTCGCTCTGGTTCAGC
c (TBX3)	CGAGCTCAGGGGCTGCA/ATAGGCGTGGTTTTCACAGG
